# Role of Computed Tomography before Transcatheter Pulmonary Valve Implantation in Patients with Dysfunctional Native Right Ventricular Outflow Tract

**DOI:** 10.3390/diagnostics13203231

**Published:** 2023-10-17

**Authors:** Paweł Gać, Agnieszka Trejtowicz-Sutor, Konrad Witkowski, Rafał Poręba

**Affiliations:** 1 Centre for Diagnostic Imaging, 4th Military Hospital, Weigla 5, PL 50-981 Wroclaw, Poland; 2 Department of Population Health, Division of Environmental Health and Occupational Medicine, Wroclaw Medical University, Mikulicza-Radeckiego 7, PL 50-368 Wroclaw, Poland; 3 Department of Internal and Occupational Diseases, Hypertension and Clinical Oncology, Wroclaw Medical University, Borowska 213, PL 50-556 Wroclaw, Poland

**Keywords:** computed tomography, right ventricular outflow tract, transcatheter pulmonary valve implantation

## Abstract

The most performed percutaneous valve replacement procedure is for the aortic valve. In recent years, there have been developments in percutaneous valve replacement methods for other valves, including the pulmonary valve. Computed tomography plays a crucial role in various stages of procedure planning and provides essential information regarding potential complications after the procedure. We present images documenting step by step how to evaluate cardiac computed tomography in the qualification procedure for transcatheter pulmonary valve implantation in patients with dysfunctional native right ventricular outflow tract.

In cases of pathological heart valve conditions, the standard treatment method is valve replacement. Valve replacement can be performed surgically or through a less invasive percutaneous approach. The most commonly performed percutaneous valve replacement procedure is for the aortic valve. In recent years, there have been developments in percutaneous valve replacement methods for other valves, including the pulmonary valve [1].

The primary indication for transcatheter pulmonary valve implantation (TPVI) is to treat dysfunction of the right ventricular outflow tract (RVOT) in patients who have undergone surgery for congenital heart defects, with the goal of preventing irreversible damage to the right ventricle (RV). Initially, TPVI was an alternative to surgery and was primarily used in patients with dysfunctional conduits connecting the right ventricle to the pulmonary artery (RV-PA conduit). However, it is now also used in patients with dysfunctional native RVOT, which has expanded the pool of eligible cases [2,3]. Computed tomography (CT) plays a crucial role in various stages of procedure planning and provides essential information regarding potential complications after the procedure.

Before TPVI, CT imaging helps in patient selection, identifying contraindications to the procedure, and choosing the appropriate device. CT images are used to assess the morphology of the RVOT and the anatomy of the main pulmonary artery (MPA) [4,5].

To reduce artifacts and optimize image quality, a three-phase administration of contrast medium is used. Three-phase contrast agent administration involves the administration of: contrast agent alone, a mixture of contrast agent and physiological saline, and physiological saline only. Optimization of the ionizing radiation dose in this type of examination is particularly important. The methods that reduce the dose of ionizing radiation include CT tube voltage reduction, ECG-monitored radiation modulation (tube current), iterative image reconstruction, deep learning-based image reconstruction and deep learning-based image denoising, reduction in the scan range (scan length), modulation of the current intensity depends on the attenuation of the radiation, heart rate control, multi-slice, and dual-source and wide-field tomography. Retrospective ECG-gated CT facilitates dynamic assessment of the RVOT and pulmonary valve in all phases of the cardiac cycle, as well as quantitative evaluation of heart chamber volumes [6].

The first piece of information obtained from CT images is the shape of the RVOT, which can take five different forms. Type I (most common, at 44.6%) is pyramidal or aneurysmal (Figure 1), type II is cylindrical, type III is an inverted pyramid, type IV is spindle-shaped, and type V is concave [4,5].

Due to the high isotropic spatial resolution in all phases and planes of the heart, CT is the most suitable method for measuring the native RVOT. Images from double-oblique reconstructions of the RVOT and PA are used for this purpose. Implantation in the native RVOT is more challenging than in the RV-PA conduit due to its larger, variable, and dynamic geometry, as well as its unpredictable compliance. Therefore, a comprehensive assessment of the native RVOT is required to determine the appropriate valve bioprosthesis. Measurements of surface areas, circumferences, and diameters at six different levels from the RVOT to the PA bifurcation are needed [5,6] (Figure 2 and Figure 3). Depending on the measurements taken, in accordance with the prosthesis manufacturer’s instructions, the appropriate size of the prosthesis is selected.

These measurements can change by up to 50% during the cardiac cycle, so it is essential to perform measurements in the end-systolic phase when the RVOT and MPA are largest, atrioventricular valves are closed, and heart chamber volumes are smallest, as well as in the end-diastolic phase when the RVOT and MPA are smallest, and heart chamber volumes are largest [4,6] (Figure 4).

Additionally, three measurements of distances between the RVOT, pulmonary valve, and MPA bifurcation are performed [4] (Figure 5).

Potential complication associated with TPVI is the compression of the coronary artery. The risk of coronary artery compression is assessed during the heart catheterization procedure by inflating a balloon in the RVOT. However, CT imaging before the procedure can also help estimate the risk of coronary artery compression. The assessment involves measuring the distance between the coronary arteries and the RVOT or conduit. A distance of less than 2 mm between the coronary arteries and the RVOT is considered to increase the risk of coronary artery compression after TPVI [4,5,6] (Figure 6).

CT examination also provides an information about vascular access and potential non-cardiac contraindications to the procedure.

## Figures and Tables

**Figure 1:**
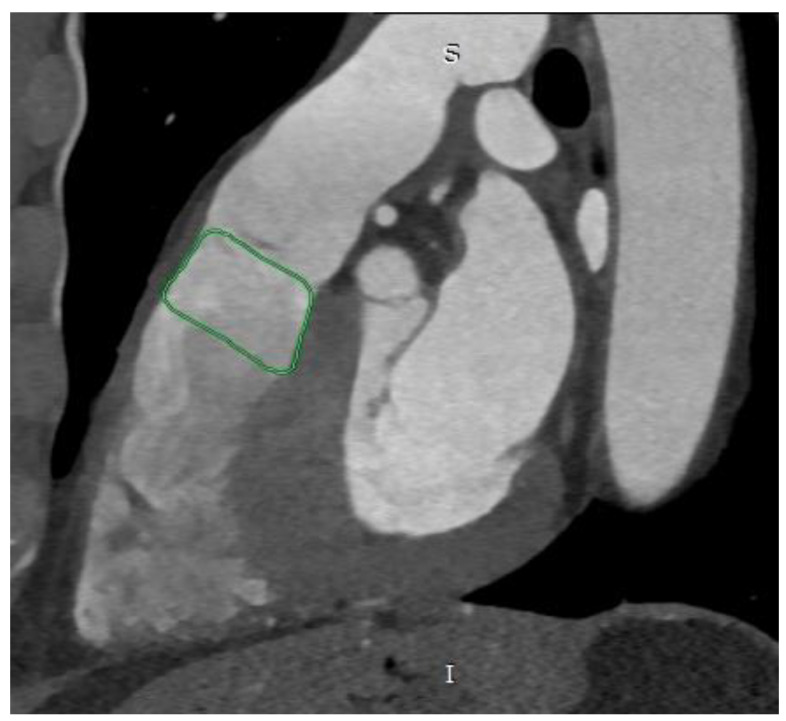
Cardiac computed tomography before transcatheter pulmonary valve implantation. MPR reconstruction. The green outline indicates the pyramidal shape of the right ventricular outflow tract. S—superior, I—inferior.

**Figure 2:**
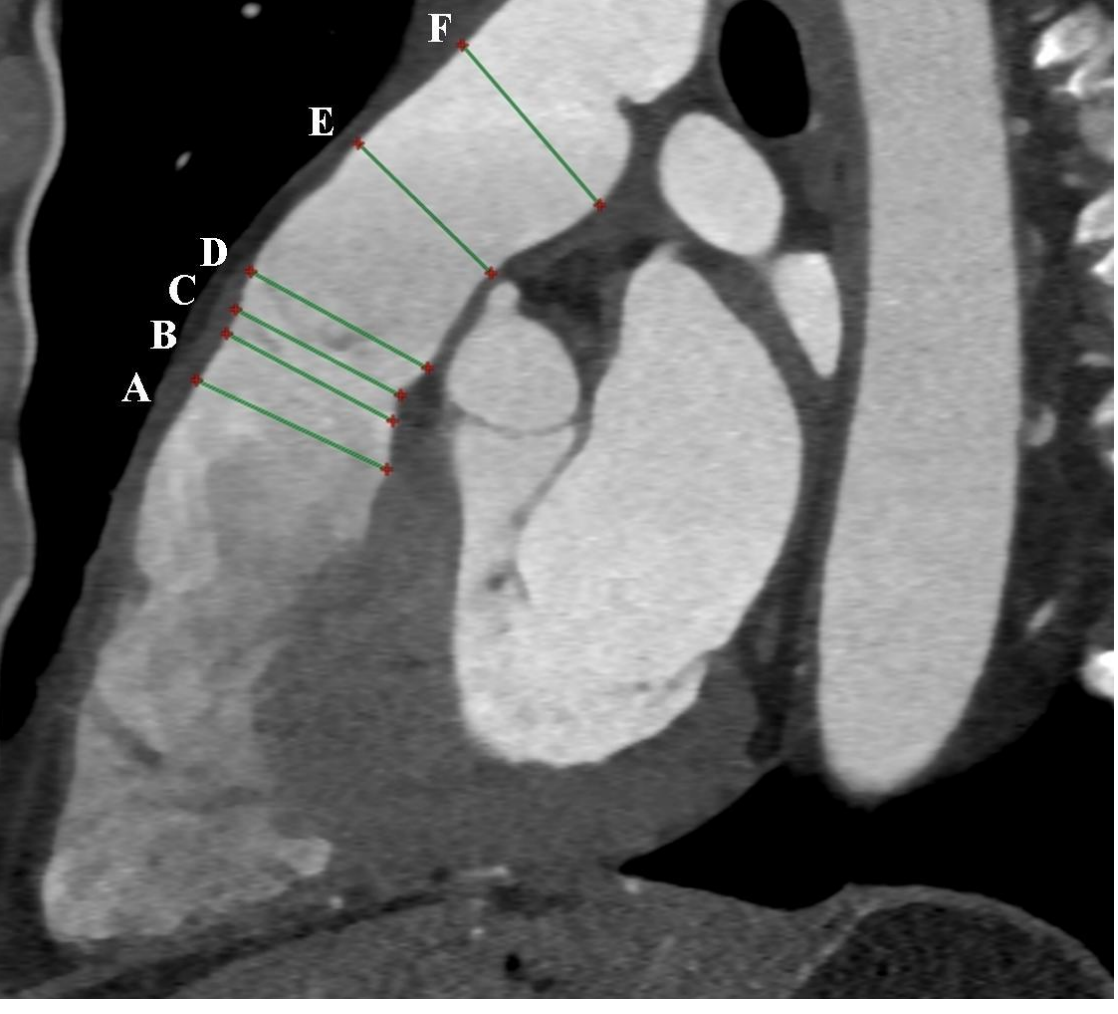
Cardiac computed tomography before transcatheter pulmonary valve implantation. MPR reconstruction. The green lines indicate 6 consecutive dimensions levels: A—level of the right ventricular outflow tract; B—level under the pulmonary valve; C—level of the pulmonary valve; D—level above the pulmonary valve; E—level of the middle part of the main pulmonary artery; F—level before the bifurcation main pulmonary artery.

**Figure 3:**
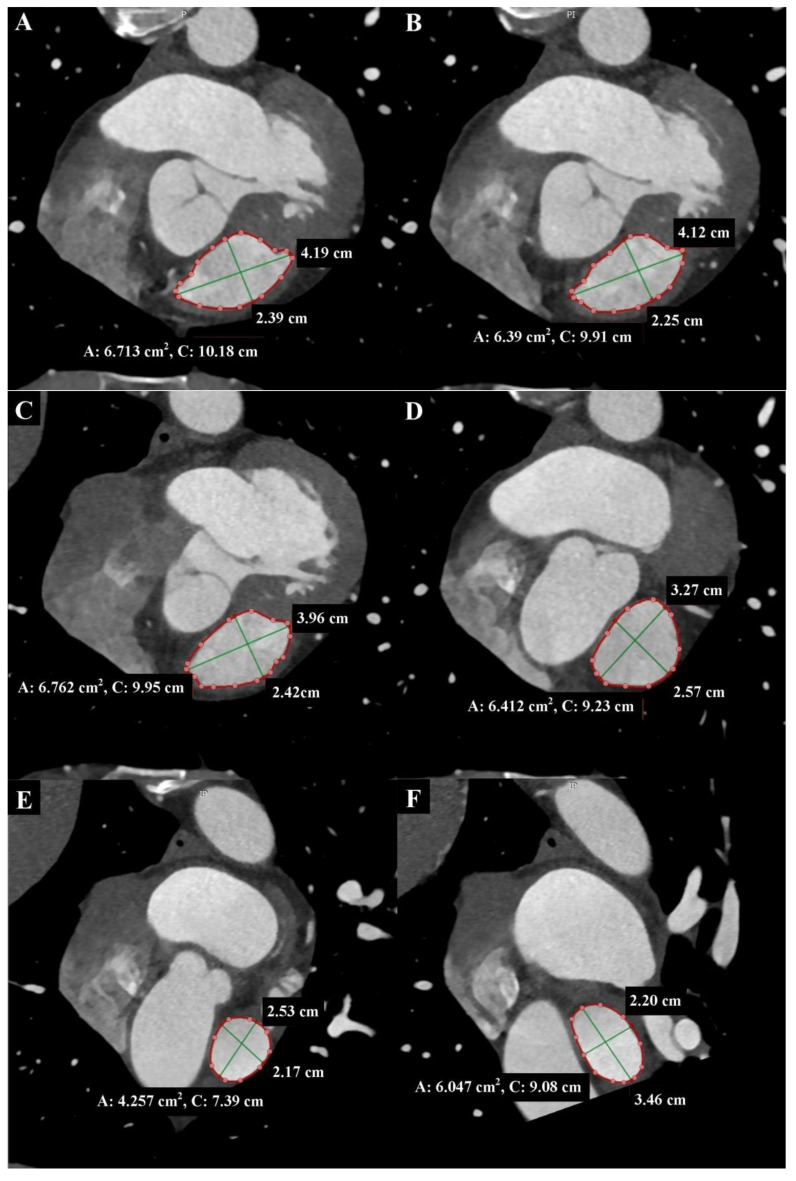
Cardiac computed tomography before transcatheter pulmonary valve implantation. MPR reconstruction. End-diastolic phase. The cross-sections shown in each Panel are taken where the green lines are in Figure 2, respectively. (**A**) Level of the right ventricular outflow tract. (**B**) Level under the pulmonary valve. The green lines indicate the longitudinal and transverse dimensions of the cross-section, and the red line indicates the cross-section outline. Cardiac computed tomography before transcatheter pulmonary valve implantation. MPR reconstruction. End-diastolic phase. The cross-sections shown in each Panel are taken where the green lines are in Figure 2, respectively. (**C**) Level of the pulmonary valve. (**D**) Level above the pulmonary valve. (**E**) Level of the middle part of the main pulmonary artery. (**F**) Level before the bifurcation main pulmonary artery. The green lines indicate the longitudinal and transverse dimensions of the cross-section, and the red line indicates the cross-section outline.

**Figure 4:**
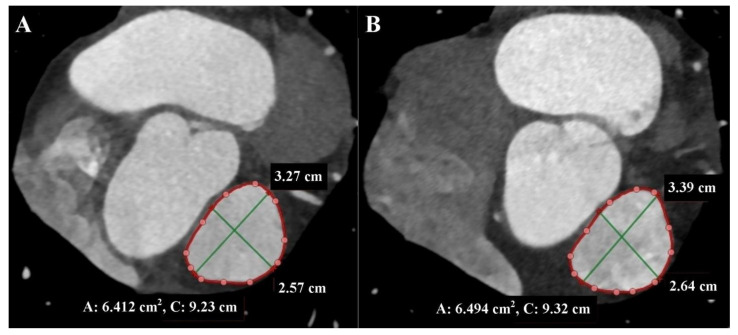
Cardiac computed tomography before transcatheter pulmonary valve implantation. MPR reconstruction Level above the pulmonary valve. (**A**) End-diastolic phase. (**B**) End-systolic phase. The green lines indicate the longitudinal and transverse dimensions of the cross-section, and the red line indicates the cross-section outline.

**Figure 5:**
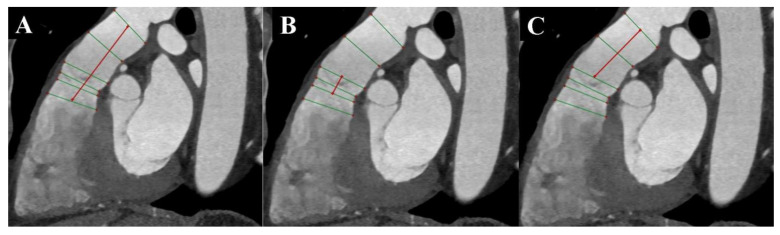
Cardiac computed tomography before transcatheter pulmonary valve implantation. MPR reconstruction. (**A**) Measurement of the distance between the level of the right ventricular outflow tract and the level before the bifurcation main pulmonary artery. (**B**) Measurement of the distance between the level under the pulmonary valve and the level above the pulmonary valve. (**C**) Measurement of the distance between the level under the pulmonary valve and the level before the bifurcation main pulmonary artery. The green lines indicate 6 consecutive dimensions levels, and red lines indicate measured distances.

**Figure 6:**
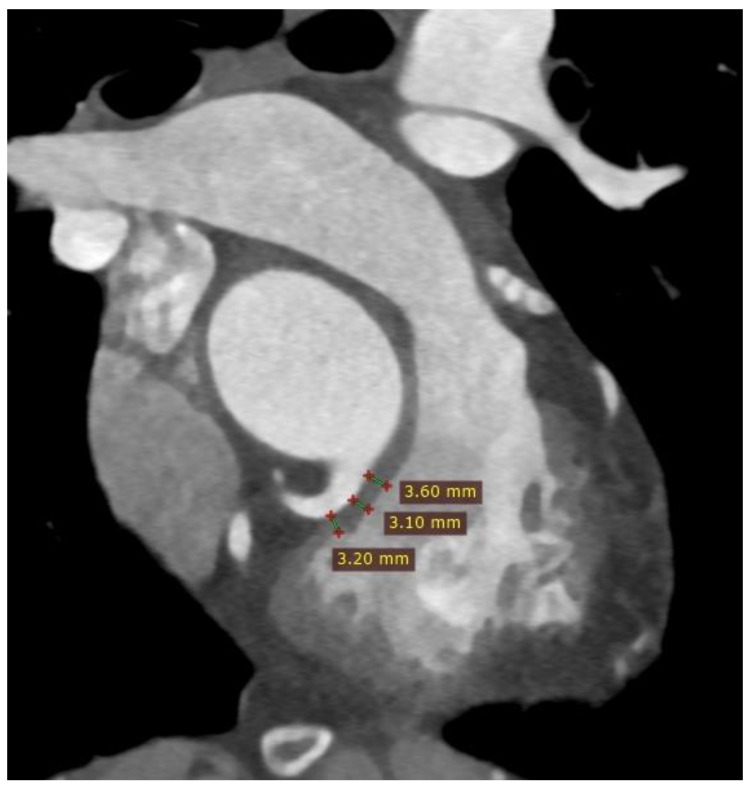
Cardiac computed tomography before transcatheter pulmonary valve implantation. MPR reconstruction. Red lines indicate the measured distances of the right ventricular outflow tract from the proximal segment of the right coronary artery.

## Data Availability

Not applicable.
